# Understanding Comorbidities in Hypermobile Ehlers-Danlos Syndrome: Could a Viral Infection Lead to a Diagnosis?

**DOI:** 10.1101/2025.10.22.25338573

**Published:** 2025-10-24

**Authors:** Megan L. Pearson, Bryan J. Laraway, Ellen R. Elias, Ganna Bilousova, Melissa A. Haendel, National Clinical Cohort Collaborative (N3C) Consortium

**Affiliations:** 1) Department of Dermatology; Gates Institute, University of Colorado Anschutz Medical Campus, Aurora CO, USA; 2) Department of Genetics; University of North Carolina, Chapel Hill NC, USA; 3) Department of Pediatrics and Genetics, Children’s Hospital Colorado, Aurora CO, USA; 4) National Clinical Cohort Collaborative (N3C) Enclave

## Abstract

Hypermobile Ehlers-Danlos Syndrome (hEDS) is a complex, underdiagnosed connective tissue disorder characterized by widespread symptoms affecting multiple organ systems. Recent clinical observations suggest that individuals with hEDS may be at increased risk for persistent symptoms following COVID-19, commonly referred to as Long COVID. Using data from over 23 million patients across the United States, we examined associations between hEDS, COVID-19 infection, Long COVID, and related chronic conditions.

We identified nearly 30,000 individuals with hEDS and found that the estimated prevalence was approximately 1 in 800, higher than previously recognized. While rates of COVID-19 infection were similar between patients with hEDS and matched controls, those with hEDS were significantly more likely to develop Long COVID. This risk was especially elevated among patients with hEDS with overlapping conditions commonly seen in post-viral syndromes, including autonomic dysfunction, immune dysregulation, and chronic fatigue. Specifically, individuals with postural orthostatic tachycardia, mast cell-related symptoms, or chronic fatigue syndrome had the highest rates of Long COVID.

Cumulative incidence analysis revealed that many patients received an hEDS diagnosis only after a COVID-19 infection, suggesting that viral illness may exacerbate or reveal previously unrecognized symptoms. Patients with hEDS also exhibited higher odds of having additional risk factors for severe or prolonged illness, including chronic lung and autoimmune conditions, depression, and cerebrovascular disease.

These findings highlight a previously unrecognized vulnerability in patients with hEDS and underscore the need for greater clinical awareness of their heightened risk for persistent post-COVID illness. Improved screening, earlier diagnosis, and integrated care pathways are urgently needed to support this complex and underserved patient population.

## Author summary

We studied patients with hypermobile Ehlers-Danlos Syndrome (hEDS), a connective tissue condition that affects joints, skin, and many body systems. This condition is often misunderstood or overlooked, leaving many people undiagnosed. During the COVID-19 pandemic, people with hEDS appeared to experience more long-term symptoms after infection, a condition often called Long COVID. Here, we analyzed the health records of millions of patients in the United States to better understand post-viral outcomes.

Patients with hEDS were more likely to be diagnosed with Long COVID compared to similar patients without hEDS. This was especially true for those who also had conditions such as chronic fatigue, immune conditions, or issues with heart rate and blood pressure regulation. In many cases, people were diagnosed with hEDS for the first time only after they had COVID-19, suggesting the virus may worsen or reveal symptoms that had been previously missed.

Our findings show that hEDS may be more common than previously thought and that such patients face higher risks after COVID-19. Greater awareness and earlier recognition of hEDS could improve care for many patients with complex, long-lasting symptoms

## Introduction

**Figure 1.**
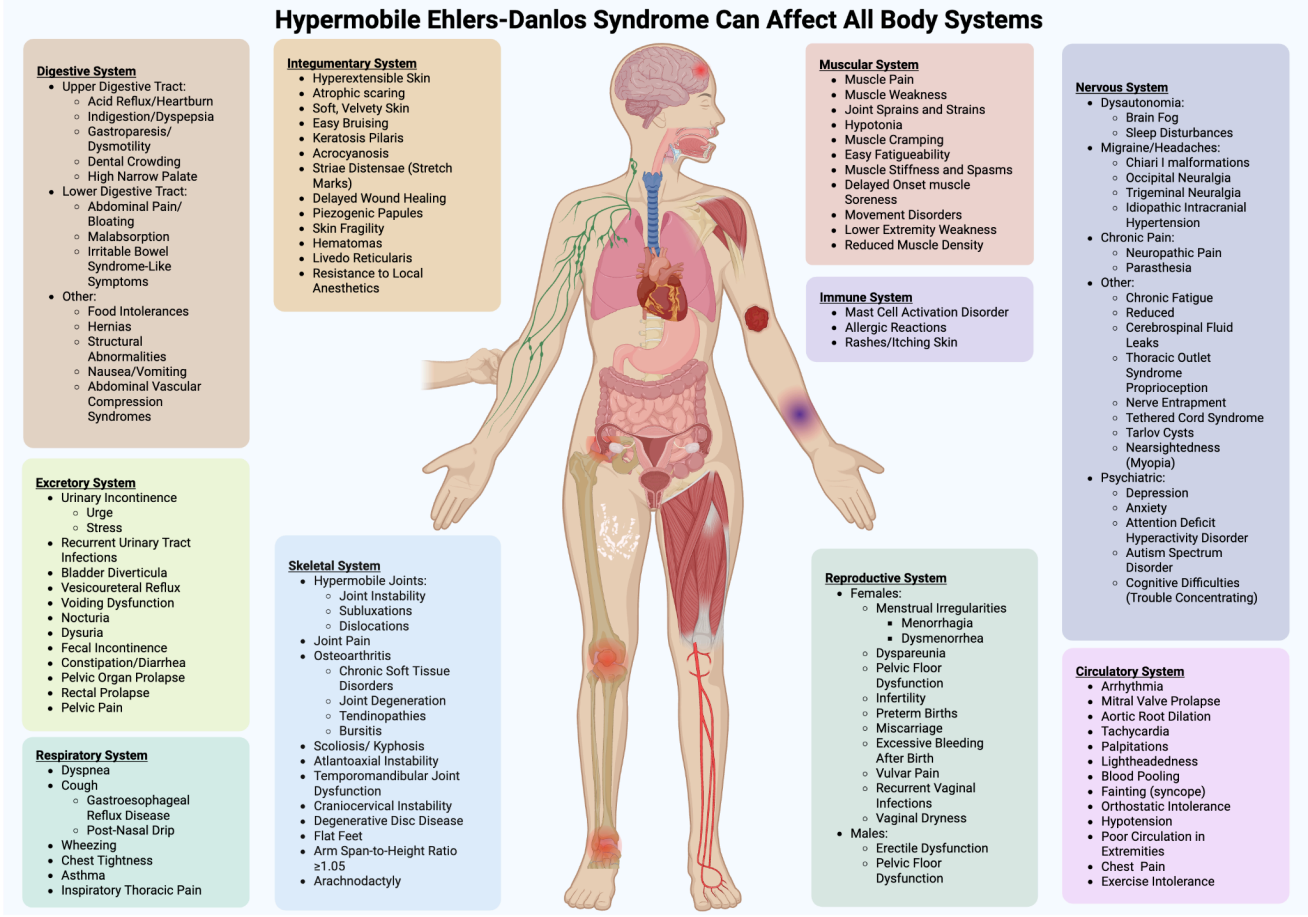
hEDS symptoms can affect all body systems. Patients with hEDS can exhibit manifestations in nearly all body systems making this complex disorder challenging to characterize and diagnose. Shown is an inclusive list of hEDS manifestations and frequently co-occurring conditions. The multisystem involvement without an apparent unifying cause frequently leads to diagnostic delays, as the underlying connective tissue disorder is not readily recognized.

Hypermobile Ehlers-Danlos Syndrome (hEDS) is the most common subtype of the Ehlers-Danlos Syndromes (EDS), a group of inherited connective tissue disorders caused by pathogenic variants affecting extracellular matrix components and collagens[1–3]. Unlike the other 13 EDS subtypes, hEDS is the only subtype with no known molecular marker and is diagnosed clinically based on skin hyper extensibility, generalized joint hypermobility, systemic features, and exclusion of other conditions[2,4]. hEDS is highly heterogeneous, with multisystem involvement that can include musculoskeletal pain, gastrointestinal dysmotility, autonomic dysfunction, and neurocognitive symptoms[5–11]. Figure 1 highlights the range of symptoms and body systems that can be affected in hEDS, which vary widely across individuals in severity and combination[11–13].

This complexity contributes to frequent misdiagnosis or diagnostic delays. hEDS symptoms commonly overlap with better-recognized disorders such as fibromyalgia, irritable bowel syndrome, and psychiatric or somatic symptom disorders[6–8,14,15]. Autonomic features, including dizziness, syncope, and tachycardia, may meet criteria for postural orthostatic tachycardia syndrome (POTS)[9,10], while neurocognitive and affective symptoms are often attributed to anxiety or ADHD. The frequent co-occurrence in patients with hEDS of mast cell activation syndrome (MCAS), a poorly defined disorder marked by episodic hypersensitivity and gastrointestinal symptoms, adds additional diagnostic ambiguity[16–19]. While patients may present with POTS and MCAS, this does not mean they automatically have a diagnosis of hEDS as these can be co-occurring but are separate conditions. We hypothesize that hEDS is not a single disorder caused by a single gene mutation, but rather multiple disorders caused by many different genetic variants, giving rise to the variable symptoms exhibited by patients with hEDS.

Joint hypermobility, a hallmark of hEDS, often diminishes with age or injury, making recognition more difficult over time[4,20–22]. Many systemic manifestations emerge or intensify after key life events such as puberty, pregnancy, or trauma, transitions often accompanied by hormonal shifts. Notably, approximately 80% of patients with hEDS are female, and mounting evidence suggests a link between hEDS manifestations and hormonal influences, particularly estrogen[23]. These hormonal factors may modulate connective tissue integrity and symptom expression, contributing to sex-based differences in prevalence and clinical presentation. Without visible hypermobility or a known genetic marker, clinicians may not suspect a connective tissue disorder. Diagnosis is often delayed by years, with patients reporting prolonged medical odysseys involving multiple specialties and inconsistent care[2,24–26]. Given this diagnostic complexity, the true prevalence of hEDS is likely underestimated.

Recent reports suggest patients with hEDS may also be at increased risk for post-acute sequelae of SARS-CoV-2 infection (COVID-19), commonly referred to as Long COVID [27–30]. This overlap may be driven by shared pathophysiologic features, including immune dysregulation (e.g., MCAS, autoimmunity), chronic inflammation, and dysautonomia (e.g., POTS). Structural tissue fragility may further predispose patients with hEDS to respiratory and vascular complications during and after COVID-19. Comorbidities common in hEDS, such as Myalgic Encephalomyelitis/Chronic Fatigue Syndrome (ME/CFS), chronic pain, and gastrointestinal dysfunction, also align with known Long COVID risk factors. Anecdotal and clinical observations suggest that SARS-CoV-2 infection may exacerbate or unmask underlying hEDS symptoms, triggering a new diagnosis.

Despite plausible biological and clinical connections, there is a lack of large-scale, population-level studies evaluating the relationship between hEDS and Long COVID. This study uses electronic health record (EHR) data from the National Clinical Cohort Collaborative (N3C) COVID-19 Enclave to examine the burden of COVID-19 and Long COVID among patients with hEDS, characterize key comorbidities, and assess whether viral infection may act as a diagnostic inflection point for previously unrecognized hEDS.

## Methods

### Data source: National Clinical Cohort Collaborative (N3C) COVID-19 enclave

This study utilized Level 2 de-identified data, including date-shifted event timestamps, the N3C COVID-19 Enclave—a centralized, harmonized repository of EHRs contributed by more than 80 U.S. clinical institutions [30] (Data Use Request: RP-582482). All patient dates are uniformly shifted by up to ±180 days within each site to protect patient privacy. As a result, temporal relationships within each patient record are preserved, but cross-patient calendar dates are not comparable.

The N3C Enclave was established in April 2020 to facilitate national COVID-19 research. It includes three primary patient groups: (1) individuals tested for SARS-CoV-2; (2) those diagnosed with COVID-19 via laboratory confirmation or clinical coding; and (3) comparison patients with at least one encounter after January 1, 2020, but no record of COVID-19 infection or exposure [31]. Retrospective data from January 1, 2018, allow evaluation of pre-pandemic health status.

As of the April 1, 2025 data release, the N3C contained over 23.5 million unique patient records, including 9.14 million confirmed COVID-19 cases. All data are harmonized to the Observational Medical Outcomes Partnership (OMOP) Common Data Model, enabling standardized querying across demographics, diagnoses, procedures, medications, labs, and clinical observations. Diagnoses from ICD-9/10-CM codes are mapped to OMOP concept IDs and stored in the condition occurrence table. Concept IDs used for this study are listed in Supplementary Table 1.

### Phenotype definitions and COVID-19/Long COVID classification

We leveraged the N3C Logic Liaison Table[31] to define COVID-19 cases, Long COVID, and related risk conditions[31]. This curated tool maps clinical phenotypes to OMOP concept sets using standardized inclusion/exclusion criteria. Supplementary Table 2 summarizes phenotypes used in this analysis. Long COVID codes were inconsistently used across time and institutions; prior to October 2021, B94.8 and non-specific codes were commonly used. As a result, both Long COVID and COVID-19 may be underrepresented in structured data.

### Cohort definition: hEDS identification

**Figure 2:**
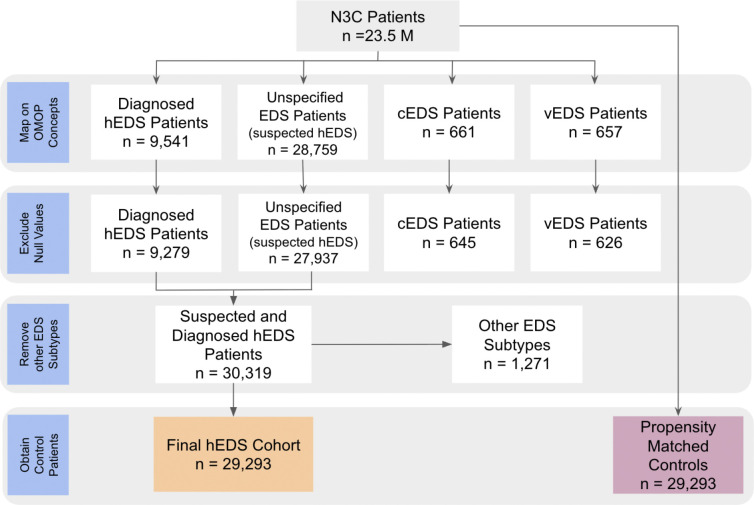
Cohort construction. Patients in the N3C database with a diagnostic code for hEDS were used to construct the cohort. Diagnosed hEDS cases were identified using the specific condition ID for hEDS, while suspected cases were identified using the condition ID for EDS with unspecified subtype. Patients with missing values for sex, age, race, or ethnicity were excluded. To minimize inclusion of other EDS subtypes, individuals with diagnostic codes for classical (cEDS) or vascular EDS (vEDS) were also excluded, yielding a final cohort of 29,293 patients. Propensity matched controls for age, sex, race, ethnicity, and clinical site were obtained from the base N3C patient population of 23.5M.

To identify patients with hEDS, we queried the N3C Data Enclave using OMOP concept IDs mapped to diagnostic codes for hEDS (Figure 2). In addition to patients explicitly diagnosed with hEDS, we also identified individuals coded with classical EDS (cEDS), vascular EDS (vEDS), and unspecified EDS. Because cEDS and vEDS are the next most common EDS subtypes after hEDS and have distinct ICD-to-OMOP mappings, we excluded individuals with these diagnoses to avoid misclassification. Given that 80–90% of all EDS cases are believed to be hEDS, we included patients with the unspecified EDS code as suspected hEDS cases. We identified 9,451 patients with a specific hEDS diagnosis and 28,759 patients with the unspecified EDS code. To ensure data completeness, we excluded patients lacking demographic information, including age, sex, race, and ethnicity. After filtering, the final diagnosed hEDS group included 9,279 patients, and the suspected hEDS group included 27,937 patients. These two groups were combined to create a final hEDS cohort of 30,319 individuals. To confirm that cEDS and vEDS cases were not inadvertently included in the final cohort, we separately identified patients with those diagnoses and retained only those with complete demographic data, 645 cEDS and 626 vEDS patients respectively. The final hEDS cohort thus consisted of 29,293 patients. At the time of analysis, the total N3C population included approximately 23.5 million patients.

### Comorbidity identification

We focused on three conditions frequently co-occurring with hEDS and Long COVID: MCAS, POTS, and ME/CFS. Patients were flagged as positive for each comorbidity if they had ≥1 recorded OMOP concept. Diagnosis dates were used to assess temporal relationships to hEDS, COVID-19, and Long COVID diagnoses. Patients could have multiple comorbidities to reflect real-world multimorbidity patterns.

### Statistical Analyses

#### Prevalence Estimation

Prevalence rates were calculated to describe the burden of hEDS, comorbidities, COVID-19 infection, and Long COVID, within the study cohort. The denominator included all individuals in the N3C Enclave with at least one clinical encounter between 2018 and 2025 and complete demographic information. Cases were identified using OMOP concepts outlined in the cohort construction and comorbid conditions. Point prevalence was calculated as the proportion of individuals meeting case criteria at any time during the study window.

#### Propensity Score Matching

To control for potential confounding by demographic and institutional differences, we constructed a propensity score–matched control cohort using the MatchIt package in R[32]. One-to-one nearest-neighbor matching without replacement was performed based on age, sex, race, and data partner (i.e., contributing clinical site). Matching on data partners was included to mitigate potential bias arising from variability in diagnostic coding practices and population demographics across institutions. The resulting control cohort comprised 29,293 individuals with no recorded diagnosis of EDS, including hEDS, cEDS, vEDS, or unspecified EDS subtypes.

#### Odds Ratios

We estimated crude odds ratios (ORs) and 95% confidence intervals (CIs) using crude patient counts to assess the association between hEDS and a range of clinical categories defined in the Logic Liaison Table[31]. All comparisons were made between the hEDS cohort and matched controls.

#### Cumulative Incidence Analyses

To assess the temporal relationships between diagnoses of hEDS, COVID-19, Long COVID, and related comorbid conditions, we performed cumulative incidence analyses using Kaplan-Meier estimators. Analyses were conducted on a cohort of individuals with known hEDS diagnoses, comorbid symptoms, and COVID-19 confirmed infection history. Dates of initial diagnoses were extracted from clinical records for hEDS, COVID-19, Long COVID, MCAS, POTS, and ME/CFS. Patients without recorded diagnosis dates for specific conditions were censored at the time of data extraction. Due to Level 2 de-identification, all dates were shifted by up to ±180 days per patient per site, though within-patient temporal order was preserved. Two sets of cumulative incidence analyses were conducted: 1) hEDS Diagnosis After COVID-19 and Long COVID. We estimated the cumulative incidence of hEDS diagnosis following the first recorded COVID-19 date. Time to event was defined as the number of days from COVID-19 diagnosis to either hEDS diagnosis or censoring. Kaplan-Meier survival functions were inverted to yield cumulative incidence, and stratified by presence or absence of comorbid diagnoses (MCAS, POTS, or ME/CFS). Group-specific 95% confidence intervals were calculated and plotted. This process was repeated for Long COVID Indications. 2) COVID-19 and Long COVID Diagnosis After hEDS. Separate cumulative incidence curves were constructed for the time from hEDS diagnosis to subsequent diagnoses of COVID-19 and Long COVID, respectively. For each condition, patients were stratified by comorbidity status—defined by the presence of any of the following diagnoses: MCAS, POTS, or ME/CFS. Time-to-event analyses were censored at the data extraction date for patients without a diagnosis of the condition of interest. All analyses were performed in Python using the lifelines package[33]. Cumulative incidence plots include shaded 95% confidence intervals for each group.

## Results

### hEDS is more prevalent than previously recorded

Using the hEDS cohort described in the Methods, we calculated a prevalence of approximately 1 in 800 patients within the N3C Enclave. This rate is substantially higher than current estimates (~1 in 3,100) but consistent with prior findings in population-based studies, such as those conducted in Wales [24].

### hEDS cohort demographics: diagnosed patients are mostly White, Non-Hispanic females aged 25–45

Table 1 presents the demographic characteristics of the hEDS cohort. The majority of diagnosed patients were female (84.56%), White (87.80%), and Non-Hispanic (90.02%). Nearly half (48.88%) were aged 25–45. These findings are consistent with previous reports on hEDS epidemiology [24]. A matched control cohort had similar demographic distributions based on propensity matching.

### Patients with hEDS have higher Long COVID rates despite similar COVID-19 infection rates

**Figure 3.**
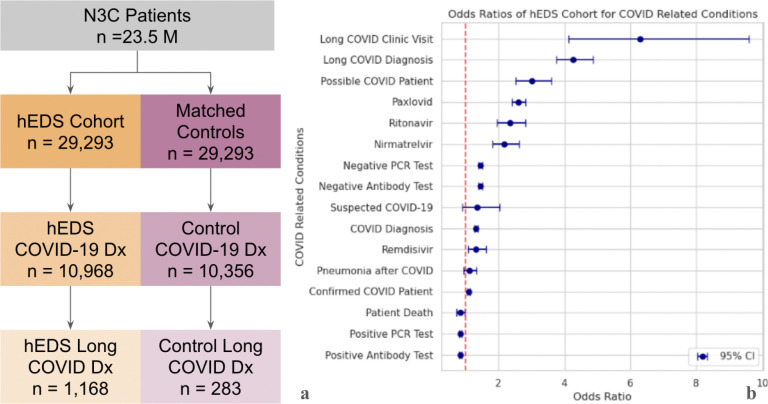
Patients with hEDS are more likely to develop Long COVID despite similar rates of COVID-19 infection **3a.** Counts of COVID-19 cases (OMOP Concept ID: 37311061) and Long COVID diagnoses (OMOP Concept ID: 705076) are shown for the hEDS cohort (orange) and matched controls (pink). **3b.** Odds ratios (ORs) with 95% confidence intervals were calculated for COVID-19–related diagnoses and medications using phenotype categories defined in the N3C Enclave (see methods). Patients with hEDS showed increased odds of Long COVID diagnoses and Long COVID clinic visits compared to controls.

To assess the relationship between hEDS and COVID-19 outcomes, we first compared rates of documented SARS-CoV-2 infection and Long COVID between patients with hEDS and propensity-matched controls, shown in Figure 3a. Using the OMOP concept for confirmed COVID-19 infection (ID:37311061), we found that 10,968 individuals (37.5%) in the hEDS cohort had evidence of COVID-19 in their EHR, compared to 10,356 (35.4%) in the matched control group, indicating similar infection rates between the two populations. However, rates of Long COVID were markedly different. Using the OMOP identifier for Long COVID (ID:705076), 1,168 (3.99%) of patients with hEDS had documentation of Long COVID, compared to only 283 (0.97%) in the control group, representing a more than fourfold increase in the hEDS population.

We then assessed COVID-19–related outcomes and treatments across both groups (Figure 3b). Odds ratio (OR) estimates confirmed that patients with hEDS had substantially higher odds of receiving an Long COVID diagnosis (OR = 4.27, 95% CI: 3.75–4.87) and attending an Long COVID specialty clinic (OR = 6.29, 95% CI: 4.12–9.60). While the odds of having a confirmed COVID-19 diagnosis were only modestly elevated in patients with hEDS (OR = 1.10, 95% CI: 1.06–1.14), the odds of being classified as a “possible COVID-19 case” based on symptoms or testing history were substantially higher (OR = 3.02, 95% CI: 2.53–3.60).

Treatment-related data revealed increased odds for the use of antiviral therapies among patients with hEDS. Specifically, the odds of receiving Paxlovid were over 2.5 times higher in patients with hEDS than controls (OR = 2.61, 95% CI: 2.41–2.82), with similar trends for ritonavir (OR = 2.32, 95% CI: 1.96–2.81) and Nirmatrelvir (OR = 2.18, 95% CI: 1.82–2.62). Remdesivir use did not differ significantly between groups (OR = 1.32, 95% CI: 1.07–1.63). In contrast, testing-related variables, such as the presence of a negative PCR or antibody test, showed no difference between groups, nor did outcomes like pneumonia or mortality following COVID-19 infection.

These results suggest that while patients with hEDS are not more likely to contract COVID-19 than demographically similar controls, they are substantially more likely to develop post-acute sequelae, to receive targeted antiviral therapies, and to seek specialized care for Long COVID.

### Long COVID is more common in patients with hEDS that also have comorbid MCAS, POTS, and ME/CFS

To investigate the relationship between common comorbidities and the likelihood of developing Long COVID in patients with hEDS, we assessed the prevalence of MCAS, POTS, and ME/CFS in patients with and without Long COVID (Table 2). Among patients with hEDS who developed Long COVID, 24.91% had a diagnosis of MCAS, compared to 10.35% of those without Long COVID. Similarly, POTS was diagnosed in 42.98% of patients with hEDS with Long COVID, compared to 25.59% of those without. ME/CFS was also more common in Long COVID-affected individuals, present in 42.29% of cases compared to 15.61% of unaffected patients with hEDS.

These associations were even more pronounced in patients with multiple co-occurring comorbidities. MCAS and POTS were jointly diagnosed in 19.09% of patients with hEDS with Long COVID, versus 5.22% of those without. MCAS and ME/CFS co-occurred in 13.96% of Long COVID patients, compared to 2.83% in those without Long COVID. Likewise, POTS and ME/CFS were present in 22.26% of Long COVID-affected patients with hEDS, versus 5.17% of those without Long COVID. Notably, 11.56% of patients with hEDS with Long COVID had all three conditions—MCAS, POTS, and ME/CFS—while only 1.67% of patients with hEDS without Long COVID exhibited this combination.

In contrast, these comorbid conditions were exceedingly rare among control patients without hEDS, regardless of Long COVID status. For example, ME/CFS was the only condition that appeared in more than 20 control patients with Long COVID (19.08%), while all other comorbidity combinations occurred in fewer than 20 individuals. These findings suggest that the presence of one or more of these comorbid conditions significantly increases the likelihood of developing Long COVID in patients with hEDS, and that multisystem involvement may play a key role in post-viral vulnerability within this population.

### Patients with hEDS are more likely to receive a diagnosis after COVID-19 and Long COVID when comorbid conditions are present

**Figure 4.**
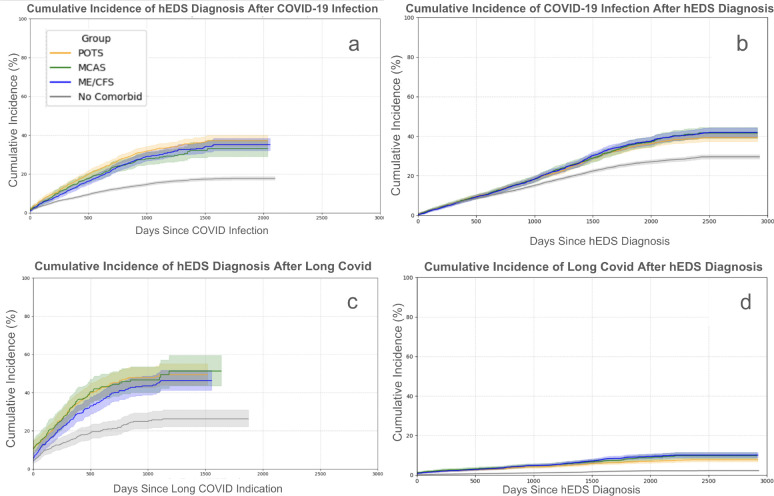
Cumulative incidence plots show patients with hEDS have higher probability of receiving a diagnosis after an acute post viral infection with complications when comorbidities are present. Cumulative incidence plots demonstrate that patients with hEDS have a higher probability of receiving a diagnosis following acute post-viral infection and experience greater risk of complications when comorbid conditions are present. Panels **4a and c** show the cumulative incidence of receiving an hEDS diagnosis after COVID-19 infection (**4a)** or Long COVID (**4c)**. Panels **4b and d** assess the cumulative incidence of developing either a COVID-19 infection (**4b)** or Long COVID (**4d)** after receiving an hEDS diagnosis. Time is measured in days from the index event (e.g., COVID-19 diagnosis or hEDS diagnosis), and cumulative incidence was estimated using the Kaplan-Meier method. Shaded regions represent 95% confidence intervals.

To investigate the temporal relationships between COVID-19, Long COVID, hEDS diagnosis, and comorbid symptom syndromes, we conducted cumulative incidence analyses using Kaplan-Meier estimators (Figure 4a–4d). These analyses show that hEDS diagnoses frequently follow COVID-19 or Long COVID onset, particularly among individuals with comorbidities suggestive of immune dysregulation (MCAS), autonomic dysfunction (POTS), or chronic fatigue (ME/CFS).

Among patients with hEDS who had COVID-19, the likelihood of receiving an hEDS diagnosis increased dramatically when comorbidities were present. As shown in Figure 4a, patients with any of the three selected comorbidities, MCAS, POTS, or ME/CFS, had a markedly elevated cumulative incidence of hEDS diagnosis following COVID-19. It does not appear that a single comorbidity contributes to increased risk and/or timing. When examining hEDS diagnosis following Long COVID onset (Figure 4c), a similar pattern emerged. These results support the hypothesis that COVID-19 or Long COVID acts as a physiological stressor that unmasks underlying connective tissue dysfunction, particularly in patients with overlapping symptom syndromes.

In reverse, we assessed the cumulative incidence of COVID-19 and Long COVID following an existing hEDS diagnosis (Figures 4b and 4d). While individuals with hEDS and comorbidities exhibited slightly elevated risk of subsequent COVID-19 or Long COVID compared to those without comorbidities, these differences were less pronounced than in the reverse temporal direction (i.e., hEDS diagnosis after COVID-19 or Long COVID).

Taken together, these findings suggest that patients with hEDS and comorbid immune, autonomic, or fatigue syndromes are not only at increased risk for Long COVID, but are also more likely to be diagnosed with hEDS after a COVID-19 infection or Long COVID presentation. This supports a model in which viral infections may trigger symptom escalation or uncover latent connective tissue dysfunction in genetically susceptible individuals.

### hEDS is associated with elevated risk factors for Long COVID and severe infection

**Figure 5.**
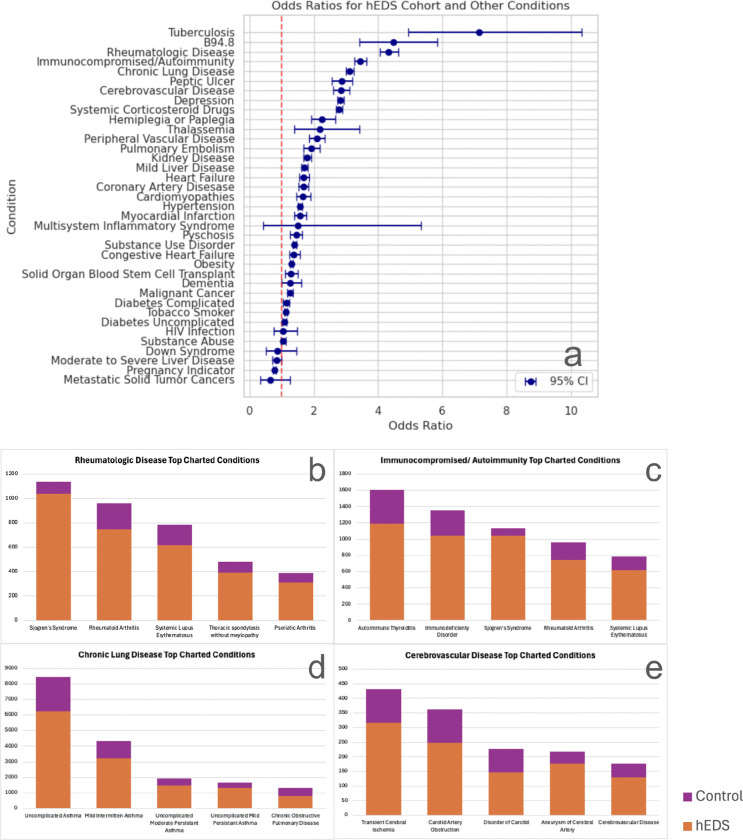
Patients with hEDS have increased covid-related category odds. **(5a)** Odds ratios (ORs) with 95% confidence intervals (CIs) are shown for COVID-19–related diagnostic categories and known risk factors for severe infection in patients with hEDS versus matched controls. Patients with hEDS demonstrated elevated odds for several infectious and chronic conditions, as well as commonly co-occurring hEDS conditions. **(5b–e)** Raw counts of top contributing diagnoses within selected enriched categories are shown: **(5b)** rheumatologic disease, **(5c)** immunocompromised/autoimmunity, **(5d)** chronic lung disease, and **(5e)** cerebrovascular disease. All patient counts fewer than 20 are reported as “<20” to protect confidentiality. Additional contributing conditions are shown in Supplementary Figure 1.

To evaluate whether patients with hEDS are more susceptible to clinical conditions that increase the risk for severe COVID-19 outcomes and Long COVID, we compared ORs for a range of pre-existing conditions between the hEDS cohort and matched controls (Figure 5a). Patients with hEDS exhibited significantly elevated odds for several diagnostic categories previously associated with adverse outcomes following COVID-19 infection.

Among the most striking associations was tuberculosis, where patients with hEDS were over seven times more likely than controls to have a diagnosis (OR = 7.14; 95% CI: 4.92–10.34). Additionally, diagnoses categorized under sequelae of infectious disease—most commonly coded using B94.8, which was frequently used to document post-viral complications including early Long COVID, were more common in the hEDS group (OR = 4.47; 95% CI: 3.41–5.84). Patients with hEDS were also substantially more likely to have diagnoses of rheumatologic diseases (OR = 4.33; 95% CI: 4.05–4.63) and autoimmune disorders (OR = 3.44; 95% CI: 3.27–3.63), both of which have been associated with increased risk of post-viral syndromes and pro-inflammatory responses to infection.

Chronic lung diseases were significantly elevated among patients with hEDS (OR = 3.12; 95% CI: 3.00–3.24), as were cerebrovascular disease (OR = 2.85; 95% CI: 2.60–3.12) and peripheral vascular disease (OR = 2.09; 95% CI: 1.86–2.35), potentially reflecting underlying connective tissue fragility in vascular and pulmonary systems. Psychosocial and treatment-related risk factors also showed increased odds, including depression (OR = 2.83; 95% CI: 2.74–2.94) and systemic corticosteroid use (OR = 2.79; 95% CI: 2.70–2.89). A smaller but statistically significant elevation was observed for thalassemia (OR = 2.19; 95% CI: 1.40–3.43). In contrast, several traditional risk factors for COVID-19 severity, such as obesity, diabetes, hypertension, liver disease, HIV, substance use disorders, and cancer did not show significant enrichment among patients with hEDS.

To further characterize the source of elevated odds, we examined condition-level data to identify the specific diagnoses contributing most strongly to each risk category (Figures 5b–e). Within the tuberculosis category, nearly all cases among patients with hEDS were vertebral tuberculosis (154 cases), compared to fewer than 20 in the control group. Similarly, the sequelae of infectious disease category was dominated by the use of ICD code B94.8, with 232 patients with hEDS having this code versus 50 controls.

The elevation in rheumatologic disease among patients with hEDS was largely driven by Sjögren’s syndrome (1,038 hEDS vs. 98 controls), rheumatoid arthritis (748 vs. 215), and systemic lupus erythematosus (618 vs. 167), with additional contributions from thoracic spondylosis and psoriatic arthritis. In the autoimmunity category, autoimmune thyroiditis (1,190 vs. 412) and immunodeficiency disorders (1,040 vs. 315) were predominant. The higher odds of chronic lung disease were primarily explained by asthma and chronic obstructive pulmonary disease (COPD). For cerebrovascular disease, transient ischemic attacks, carotid artery obstruction, and cerebral aneurysms were among the most common diagnoses.

These findings reveal that patients with hEDS carry a heightened burden of pre-existing conditions linked to poor viral outcomes and suggest a greater biological vulnerability to severe infection and persistent post-viral syndromes such as Long COVID. Additional condition-level details can be found in Supplementary Figure 1.

## Discussion

In this study, we identified a prevalence of hEDS in the N3C cohort of approximately 1 in 800 individuals, substantially higher than prior estimates, which aligns with recent work by Demmler et al.[24]. To our knowledge, this represents the first large-scale, multi-site characterization of hEDS in the United States using real-world EHR data. The increased prevalence may reflect growing clinical recognition of hEDS, heightened healthcare engagement during the COVID-19 pandemic, or a combination of both. Given the complexity and under-recognition of hEDS, it is likely that the true prevalence is even higher, reinforcing the need for increased clinical awareness, diagnostic support tools, and further prevalence studies in other populations. Further characterization of the hEDS cohort revealed a common demographic composition of White, non-Hispanic females between the ages of 25–45, consistent with prior studies[24]. While this likely reflects real patterns of disease distribution, it may also reveal disparities in access to specialty care and diagnosis. Further work is needed to understand demographic differences in symptom presentation, care-seeking behavior, and clinical recognition, as well as to identify and support underserved populations who may be at risk for missed diagnosis.

In examining the relationship between hEDS, COVID-19, and long COVID, we found that while rates of confirmed COVID-19 infection were similar between patients with hEDS and matched controls, patients with hEDS exhibited significantly higher odds of Long COVID diagnoses and Long COVID-related clinic visits. This was true even despite the known underutilization of Long COVID-specific diagnostic codes across clinical sites. Notably, we also observed elevated use of ICD-10 code B94.8—”sequelae of infectious disease”—in patients with hEDS, which was widely used as a proxy for Long COVID prior to the release of a dedicated Long COVID code (U09.9) in late 2021. Together, these findings suggest that patients with hEDS are particularly vulnerable to developing Long COVID after a COVID-19 infection, likely due to symptom exacerbation or underlying pathophysiological overlap.

We also found that patients with hEDS were more likely to receive outpatient treatments for COVID-19 such as Paxlovid (which contains Ritonavir and Nirmatrelvir), but not Remdesivir, which is administered intravenously and typically used in inpatient settings. This pattern may reflect a greater likelihood of patients with hEDS experiencing moderate COVID-19 complications warranting medical attention and pharmacologic intervention, but not hospitalization. It may also reflect provider preferences or accessibility factors, as oral treatments are generally easier to administer than IV therapies.

Comorbid conditions commonly observed in hEDS, including MCAS, POTS, and ME/CFS, were significantly more prevalent in patients with hEDS with Long COVID than in those without. While these comorbidities were not more common in patients with hEDS who contracted COVID-19 compared to those who did not, it is possible that some COVID-19 infections were unrecorded due to home testing or incomplete charting. Notably, cumulative incidence analyses revealed that hEDS diagnoses were more likely to occur after a COVID-19 or Long COVID diagnosis, particularly among patients with MCAS and POTS. This may indicate that viral infections act as unmasking events, prompting deeper clinical investigation and eventual hEDS recognition. For example, post-viral exacerbation of autonomic symptoms or immune dysregulation may lead clinicians to consider diagnoses such as POTS or MCAS, which in turn can reveal the underlying connective tissue disorder.

Nearly half of all patients with hEDS with MCAS who were later diagnosed with hEDS received that diagnosis after a COVID-19 infection. These findings support recent hypotheses that hypermobile connective tissue disorders may predispose individuals to immune dysregulation through mast cell activation, potentially contributing to both hEDS symptomatology and Long COVID risk[32]. A similar trajectory was observed for patients with POTS, suggesting that post-viral autonomic dysfunction may also act as a diagnostic trigger for hEDS. Conversely, cumulative incidence of COVID-19 or Long COVID after an hEDS diagnosis was lower, suggesting that while hEDS may not increase susceptibility to infection, it likely increases the risk of developing prolonged post-viral complications.

In addition to Long COVID-specific vulnerabilities, we observed significantly elevated odds for a range of chronic and infectious conditions in the hEDS cohort, including tuberculosis, rheumatologic disease, autoimmune disease, chronic lung disease, cerebrovascular disease, depression, systemic corticosteroid use, thalassemia, and peripheral vascular disease. Some of these, such as autoimmune conditions and depression, are well-documented hEDS comorbidities. Others, like tuberculosis and peptic ulcers, are less commonly reported. The elevated odds of tuberculosis, for instance, may reflect increased susceptibility due to immune dysregulation or preexisting lung pathology in patients with hEDS. Similarly, the observed elevation in peptic ulcers, which are not typically associated with hEDS, warrants further study. The increased use of systemic corticosteroids is also notable, given their known adverse effects on connective tissue integrity; such treatments may precede hEDS diagnosis or exacerbate undiagnosed symptoms, prompting eventual diagnostic workup. Finally, the elevated odds of hemiplegia or paraplegia in the hEDS population may reflect neurological complications stemming from central nervous system fragility or spinal instability, which are increasingly recognized as potential hEDS manifestations.

## Conclusions

This study presents the largest real-world characterization of hEDS in the United States to date, using a harmonized, multi-institutional electronic health record dataset. We estimate the prevalence of hEDS to be approximately 1 in 800 individuals, suggesting the condition is far more common than previously believed and remains substantially underdiagnosed.

Patients with hEDS demonstrated significantly elevated rates of comorbid conditions, particularly MCAS, POTS, and ME/CFS, which were also associated with increased likelihood of developing Long COVID. ORs for other infectious, rheumatologic, and neurovascular conditions were also elevated, underscoring the systemic complexity of hEDS and its potential interactions with immune and autonomic pathways.

This data supports the hypothesis that stress from immune activation after viral infections such as Long COVID in this study, often leads patients with an underlying connective tissue disorder to receive a diagnosis after symptom exacerbation post viral infection. Cumulative incidence analyses revealed that many hEDS diagnoses occurred following COVID-19 or Long COVID diagnoses, suggesting that viral illness may unmask previously unrecognized hEDS symptoms. These findings emphasize the need for integrated, multidisciplinary care models for individuals with hEDS, particularly in the context of post-viral syndromes like Long COVID. Greater clinical awareness, improved diagnostic strategies, and further investigation into the pathophysiological overlap between hEDS and viral infections are essential to ensure timely diagnosis, management, and support for this vulnerable patient population.

## Supplementary Material

Supplement 1

## Figures and Tables

**Table 1.** hEDS Cohort Demographics. hEDS patient population in N3C is majority white, non-hispanic, females, with an age range of 25–45 primarily. American Indian or Alaska Native = AI/AN. Native Hawaiian or Other Pacific Islander = NH/PI.

hEDS Cohort Demographics	n = 29,293 (%)
**Sex**	
Female	24769 (84.56)
Male	3758 (12.83)
Other/Unknown	766 (2.61)
**Ethnicity**	
Non-Hispanic	26371 (90.02)
Hispanic	1638 (5.59)
Other/Unknown	1284 (4.38)
**Race**	
White	25718 (87.80)
Black	802 (7.64)
Asian	299 (1.02)
AI/AN	195 (0.67)
NH/PI	42 (0.14)
**Age**	
0–18	2412 (8.23)
18–25	4414 (15.07)
26–35	8098 (27.64)
36–45	6221 (21.24)
46–55	4069 (13.89)
56–65	2395 (8.18)
66–75	1221 (4.17)
76–100	463 (1.58)

**Table 2.** Increased Prevalence of Comorbid Conditions in patients with hEDS who Develop Long COVID. Raw counts and corresponding prevalence rates (in parentheses) are reported for commonly recognized comorbid conditions in hEDS (29,293) and developing Long COVID (1451). For condition combinations, raw counts and prevalence rates (in parentheses) are also shown. To protect patient confidentiality, cell counts fewer than 20 are indicated as <20. Prevalence of comorbid conditions is higher in patients with hEDS who develop Long COVID indicating that when patients with hEDS have any of these comorbidities, it may be a risk factor for developing Long COVID.

hEDS Status	Long COVID Status	MCAS	POTS	ME/CFS	MCAS and POTS	MCAS and ME/CFS	POTS and ME/CFS	MCAS, POTS, and ME/CFS
*Diagnosed*	Unaffected	2649 (10.35)	5913 (25.59)	4121 (15.61)	1463 (5.22)	793 (2.83)	1448 (5.17)	467 (1.67)
*Diagnosed*	Affected	291 (24.91)	502 (42.98)	494 (42.29)	223 (19.09)	163 (13.96)	260 (22.26)	135 (11.56)
*Control*	Affected	<20	<20	54 (19.08)	<20	<20	<20	<20
*Control*	Unaffected	27 (0.09)	120 (0.41)	705 (2.15)	<20	<20	26 (0.09)	<20
