# Supplementary material for: Understanding Comorbidities in Hypermobile Ehlers-Danlos Syndrome: Could a Viral Infection Lead to a Diagnosis?
Source: medRxiv. 2025 Oct 24:2025.10.22.25338573. Preprint. [Version 1] doi: 10.1101/2025.10.22.25338573 (PMC12633589; doi:10.1101/2025.10.22.25338573)
Supplement: Supplement 1 [file media-1.pdf]

Supplementary

Supplementary Table 1: Concept IDs for Cohort Criteria

Label	Type of Concept ID	OMOP ID
Hypermobile Ehlers-Danlos Syndrome	Condition	4148925
General Ehlers-Danlos Syndrome	Condition	79145
Vascular Ehlers-Danlos Syndrome	Condition	4062070
Classical Ehlers-Danlos Syndrome	Condition	37395762
MCAS	Condition	37309640
POTS	Condition	4159659
ME/CFS	Condition	432738
COVID-19	Condition	37311061
Post-acute COVID-19 (Long COVID)	Condition	705076

Supplementary Table 2: Logic Liaison Conditions [31]

Logic Liaison Table Condition	Codeset IDs
Obesity	772561500
Kidney Disease	1000049286
Pregnancy	101607593
Rheumatologic Disease	437151010
Tobacco Smoker	166730099
Solid Organ Blood Stem Cell Transplant	225621426
Substance Use Disorder	1000043391
Long COVID Clinic Visit	1000032668
Suspected Covid-19	229223655
B94.8	783054135

Cardiomyopathies	175678032
Cerebrovascular Disease	937546319
Chronic Lung Disease	674884280
Congestive Heart Failure	1000044269
Coronary Artery Disease	313241620
Dementia	963389709
Depression	963389709
Diabetes Complicated	263424204
Diabetes Uncomplicated	689624081
Down Syndrome	859831250
Heart Failure	1000038723
Hemiplegia or Paraplegia	174172884
HIV Infection	490831143
Hypertension	211352892
COVID Diagnosis	1000012899
Long COVID Diagnosis	1000042623
Multisystem Inflammatory Syndrome	200799555
Pneumonia due to COVID	698347441
Malignant Cancer	1000073908
Metastatic Solid Tumor Cancers	1000080916
Mild Liver Disease	17655741
Moderate to Severe Liver Disease	285109253
Myocardial Infarctions	944073846
Autoimmune Disease/Immunodeficiency	1000079426
Peptic Ulcer	1000089753
Peripheral Vascular Disease	1000020459
Psychosis	361905128

Pulmonary Embolism	503985771
Sickle Cell Disease	1000028033
Substance Abuse	606624084
Thalassemia	1000047917
Tuberculosis	772103963
Nirmatrelvir	399252964
Ritonavir	329050933
Paxlovid	123908016
Remdesivir	719693192
N3C Corticosteroids for Systemic Use	363331988
Antibody Positive	871267146, 1000005668
Antibody Negative	871267146, 1000061534
PCR AG Positive	1000019497, 1000005668
PCR AG Negative	1000019497, 1000061534

Supplementary Figure 1:

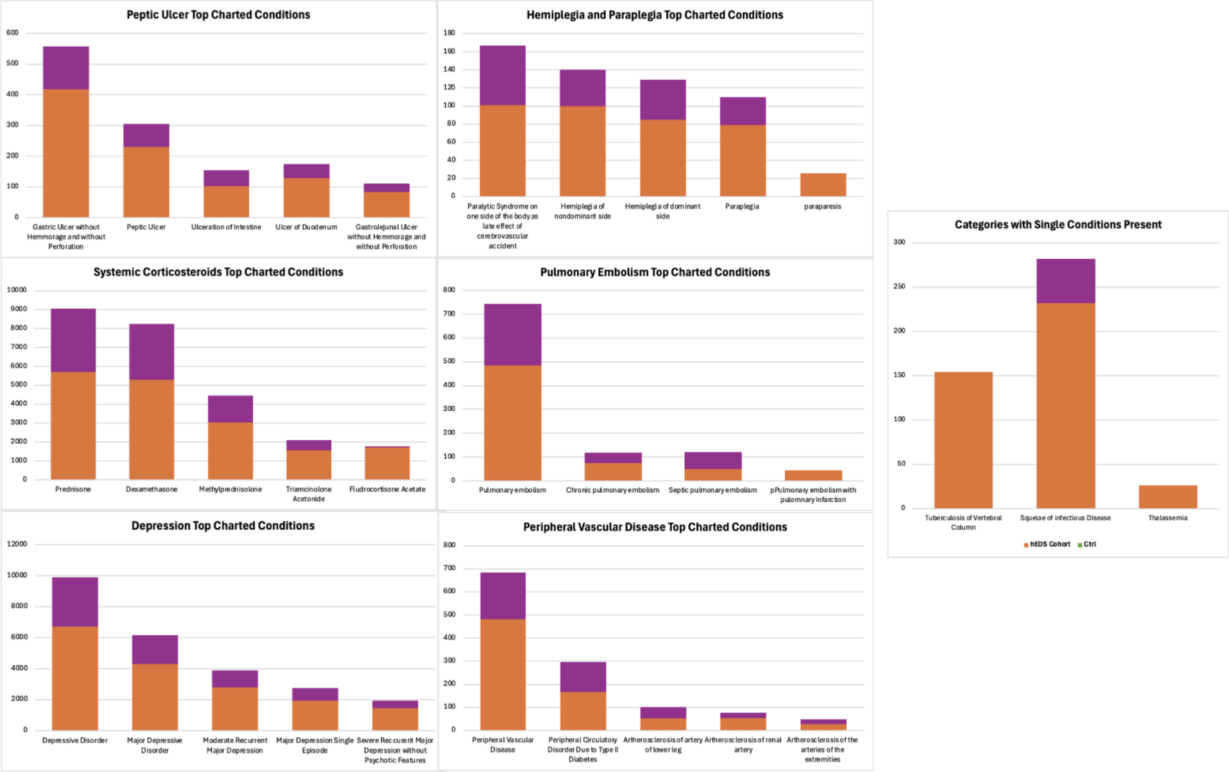

Supplemental Methods: Prevalence Calculation

Point prevalence rates were calculated to quantify the burden of hypermobile Ehlers-Danlos syndrome (hEDS), associated comorbidities, COVID-19 infection, and Long COVID within the study cohort.

The **denominator** for all prevalence estimates included all individuals in the N3C Enclave with at least one clinical encounter between January 1, 2018, and May 1, 2025, and complete demographic data (sex, age, race, and ethnicity). The **numerators** were defined by the number of individuals meeting case criteria for hEDS, each comorbidity, COVID-19, or Long COVID, based on OMOP concept sets described in the cohort construction and comorbidity definitions.

This approach captures the proportion of patients ever diagnosed with the condition during the study period, irrespective of current status.

Supplementary Table 3. Comorbid Conditions Segmented by Covid Indication.

hEDS Status	*COVID Status*	*MCAS*	*POTS*	*ME/CFS*	*MCAS and POTS*	*MCAS and ME/CFS*	*POTS and ME/CFS*	*MCAS, POTS, and ME/CFS*

Diagnosed	Unaffected	1762 (9.22)	3974 (20.79)	2710 (14.18)	1010 (5.28)	515 (2.69)	982 (5.14)	329 (1.72)
Diagnosed	Affected	1266 (11.43)	2623 (23.69)	2006 (18.12)	737 (6.66)	468 (4.23)	772 (6.97)	285 (2.57)
Control	Affected	<20	61 (0.57)	340 (3.17)	<20	<20	<20	<20
Control	Unaffected	20	87 (0.44)	420 (2.15)	<20	<20	<20	<20
